# Physician-Modified Cook Zenith Alpha Thoracic Endovascular Graft with Preservation of Active Fixation Barbs: A Step-by-Step Technical Guide

**DOI:** 10.3390/jcm15166293

**Published:** 2026-08-14

**Authors:** Emiel W. M. Huistra, Wajdi Alrawi, Ignace F. J. Tielliu, Samuel Saers, Clark J. Zeebregts, Robert C. Lind

**Affiliations:** 1 Division of Vascular Surgery, Department of Surgery, University Medical Center Groningen, University of Groningen, 9700 RB Groningen, The Netherlands; w.m.huistra@umcg.nl (E.W.M.H.); i.f.j.tielliu@umcg.nl (I.F.J.T.); c.j.a.m.zeebregts@umcg.nl (C.J.Z.); 2 Department of Thoracic and Vascular Surgery, Linköping University Hospital, 581 85 Linköping, Sweden; wajdi.alrawi@regionostergotland.se (W.A.); samuel.saers@regionostergotland.se (S.S.); 3 Department of Health, Medicine and Caring Sciences, Linköping University, 581 83 Linköping, Sweden

**Keywords:** physician-modified endograft, PMEG, Zenith Alpha, fenestrated endovascular aneurysm repair, active fixation barbs, complex abdominal aortic aneurysm keyword

## Abstract

Physician-modified endografts (PMEGs) represent an important treatment option for urgent and semi-urgent complex abdominal aortic aneurysms (cAAAs). The Zenith Alpha Thoracic Endovascular Graft (Cook Medical, Bloomington, IN, USA) is a common choice for PMEGs due to its low strut interference, albeit at the cost of requiring removal of the proximal fixation barbs for resheathing—a process that remains technically challenging. The current article details a step-by-step approach on how to modify an Alpha thoracic endograft without requiring removal of the proximal barbs. The Zenith Alpha Thoracic Endovascular Graft is fully unsheathed on a sterile back-table and completely detached from the delivery system by removing the blue rotational handle. The grey positioner is removed from the introducer sheath. Next, a 0.018-inch guidewire is introduced distally through the inner positioner and retrieved via the exposed grey handle to function as a trigger wire. Following the creation and reinforcement of the fenestrations, circular diameter-reducing ties are constructed and secured using an insertion tool. The endograft is placed back on the delivery system and the guidewire is passed through the endograft fabric at the distal end and through the insertion tool, which is then removed. At the proximal end, the trigger wire is again passed through the endograft fabric, and both the proximal bare alignment stent and the distal end of the endograft are secured to the delivery system using 2-0 Prolene sutures (Ethicon Inc., Somerville, NJ, USA). The endograft is subsequently resheathed through the distal end of the introducer sheath using a tourniquet-assisted resheathing technique and a cut-off tip from an introducer sheath to guide the endograft’s passage through the valve. Using the current standardized modification protocol, a PMEG can be constructed using the Zenith Alpha Thoracic Endovascular Graft while preserving the active fixation barbs for the treatment of cAAAs.

## 1. Introduction

The term physician-modified endograft (PMEG) was introduced by Starnes et al. in 2012 to describe the process of modifying an off-the-shelf endograft to suit patient-specific anatomy by creating fenestrations and/or branches to treat complex abdominal aortic aneurysms (cAAAs) [1]. Initial experience with PMEG was driven by a lack of access to custom-made devices (CMDs) and as CMDs have become broadly available, PMEG use has declined [2]. However, promising results with PMEG from multicentre prospective trials and large retrospective studies, combined with the manufacturing time of CMDs and their continued limited availability in several geographical regions, have resulted in continued interest in the use of PMEG [3,4,5]. Particularly in urgent and semi-urgent cAAA cases, where waiting for a CMD is considered unreasonable, PMEG was considered to be a treatment option comparable to off-the-shelf branched endografts and exceeding parallel stenting techniques in a survey comprising 227 vascular specialists from 30 countries [6].

PMEG, however, has important limitations. Most notably it falls outside the manufacturers’ intended use of the endografts and modification methods are not standardized across centres, with many different technical approaches and devices used [7,8]. Device choice plays an important role in PMEGs [9]. PMEGs for cAAA using different thoracic and bifurcated abdominal endografts, including a recently introduced dedicated PMEG system (TREO FIT, Terumo Aortic, Inchinnan, UK), have been described [10]. Two of the most commonly reported used devices for PMEG treatment of cAAAs are the Zenith TX2 Dissection Endovascular Graft (Cook Medical, Bloomington, IN, USA) and Zenith Alpha Thoracic Endovascular Graft (Cook Medical) [4]. Both platforms can also be used in the thoracic aorta and have distinct advantages and disadvantages, requiring different technical steps for PMEG construction. Specifically, the Alpha Thoracic Endovascular Graft provides larger inter-strut spaces for fenestration placement, decreasing the likelihood of fenestration–strut interference compared to the TX2 [9,11]. However, conventional PMEG construction using the Alpha Thoracic Endovascular Graft platform generally requires removal of the proximal fixation barbs before resheathing. This process may damage the endograft fabric and eliminates the active fixation mechanism incorporated into the original device design [12,13]. The current article details a standardized protocol for modifying an Alpha Thoracic Endovascular Graft for use in cAAA endovascular repair while preserving the proximal fixation barbs. In addition, the technique allows for the incorporation of a guidewire as a trigger wire for diameter-reducing ties (DRTs), with the possibility of adding two more guidewires (e.g., as precannulated wires) when required.

## 2. Technique

### 2.1. Instruments and Materials

Table 1 summarizes the minimum required instruments, materials and sutures.

### 2.2. Deployment and Removal of the Endograft from the Delivery System

**Figure 1:**
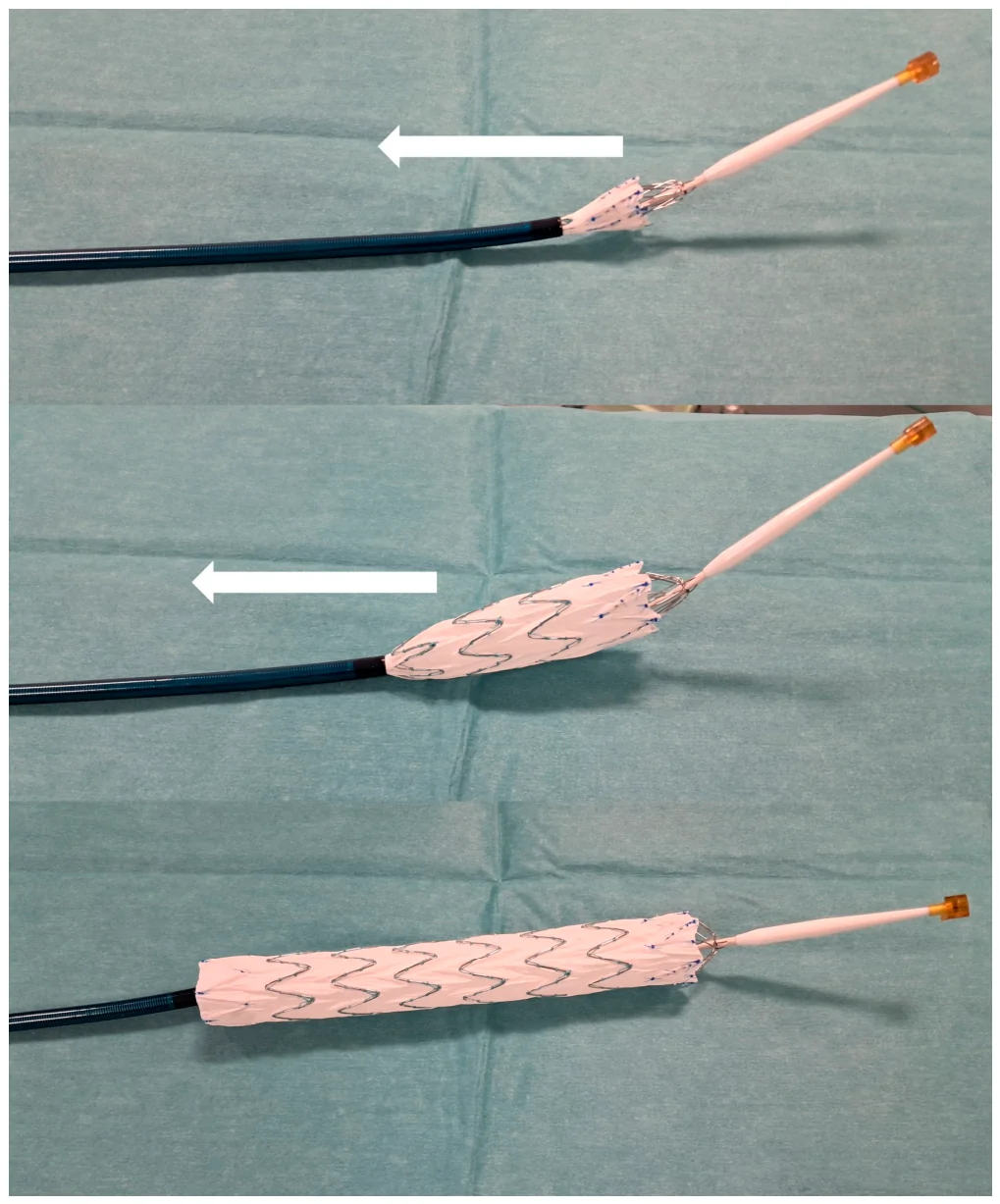
Fully expose the endograft by retracting the Flexor introducer sheath backwards (white arrow) whilst holding the grey positioner in place.

**Figure 2:**
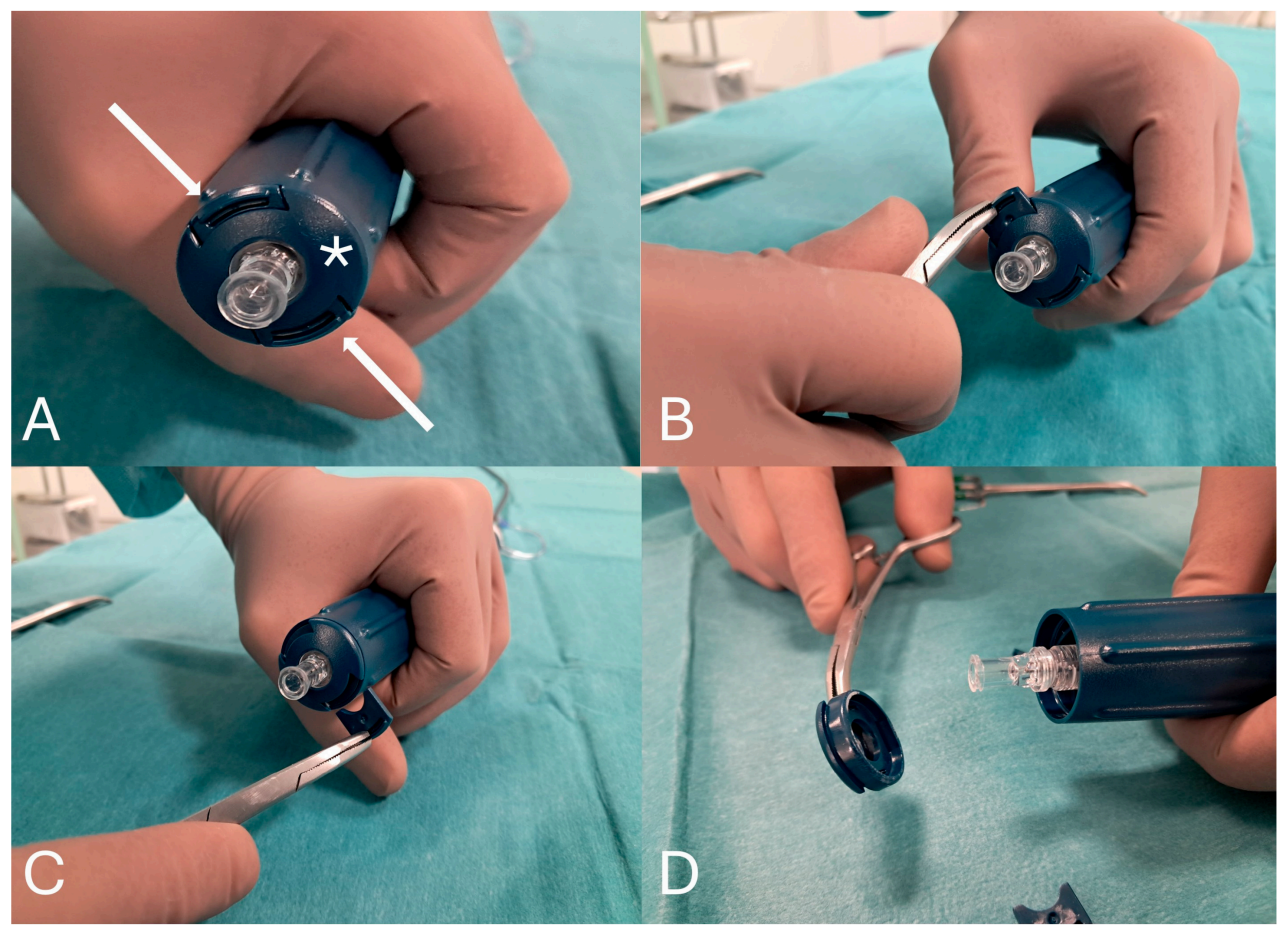
To remove the blue rotation handle, identify both back-end clips ((**A**); white arrows) and the back-end cap ((**A**); *). Remove both back-end clips (**B**,**C**) and back-end cap (**D**).

**Figure 3:**
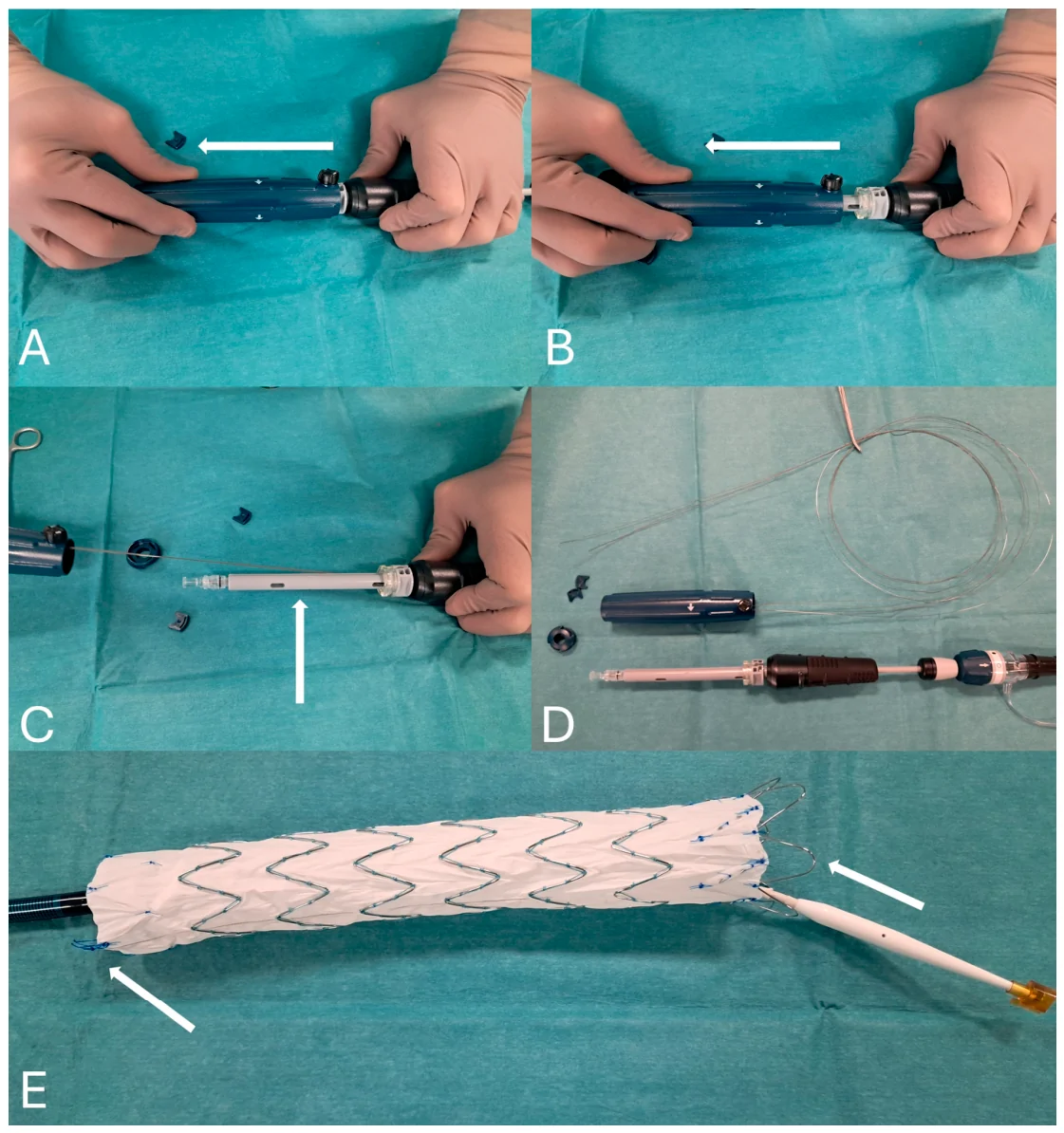
After both back-end clips and back-end cap are removed, separate the blue rotation handle from the system by pulling it backwards ((**A**,**B**); direction of the arrow) exposing the grey handle on the inside ((**C**); white arrow). The blue rotational handle with release wires should be completely removed (**D**) thereby releasing the proximal and distal fixation points of the endograft ((**E**); white arrows). The endograft can now be removed from the delivery system.

The Zenith Alpha Thoracic Endovascular Graft (proximal component) is deployed in standard fashion on a sterile back-table by retracting the outer Flexor introducer sheath over the grey positioner until the endograft is fully exposed (Figure 1). To fully deploy and remove the endograft and subsequently prepare the delivery system for modification, the blue rotation handle is removed. This is done by first removing both back-end clips and the back-end cap using forceps (Figure 2A–D), allowing for the blue rotation handle to be pulled completely away from the system, exposing the grey handle on the inside (Figure 3A–D). As the release wires are attached to the blue rotation handle, removing the blue rotation handle will release all endograft fixation points (Figure 3E). The endograft can now be fully removed from the delivery system. Care needs to be taken when manipulating the endograft as the barbs on the proximal sealing stent can cause injury to the physician and subsequently affect the sterility of the endograft.

### 2.3. Fenestrating the Endograft

Various techniques have been described for creating and reinforcing the fenestrations. A printed or drawn template can be used and positioned over the endograft to mark and subsequently create the fenestrations using a battery-powered handheld high-temperature cautery device. Alternatively, Automated Laser Modification of the Endograft (ALMEG) can be used to facilitate fenestration planning and creation, but the availability of this technique is currently limited. Both methods have previously been described in detail elsewhere [14,15,16,17].

The fenestrations are reinforced using a double loop of En-Snare (Merit Medical Systems, Inc., South Jordan, UT, USA) secured with an interlocking continuous Gore-Tex CV-6 (W.L. Gore & Associates, Flagstaff, AZ, USA) suture, with high suture density. These techniques have been previously described in more detail and are not device-specific [16,18].

### 2.4. Addition of a Trigger Wire

**Figure 4:**
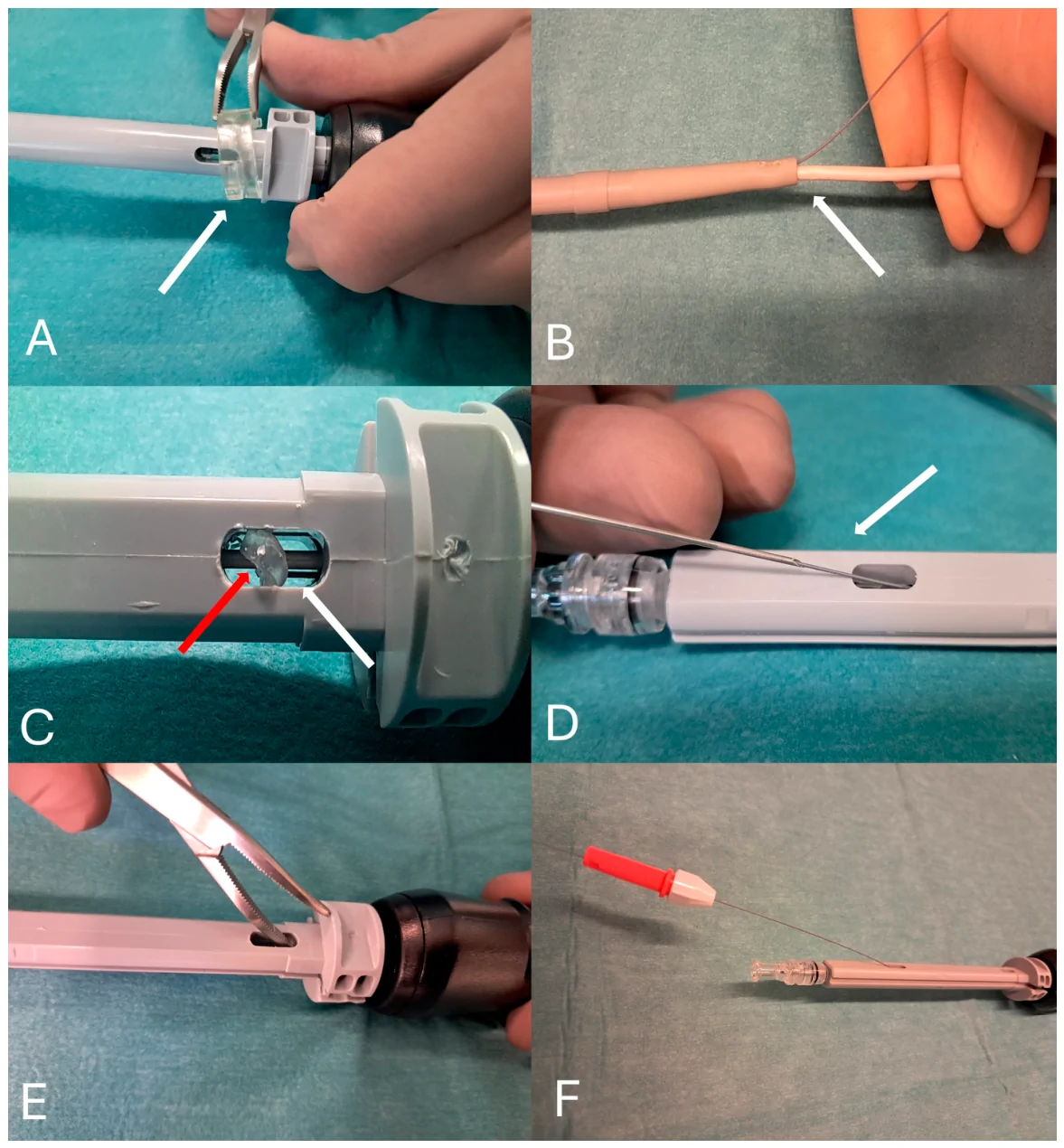
Prior to introducing the guidewire, the rubber stop is removed from the grey handle ((**A**); white arrow). The back-end of a 0.018-inch guidewire is introduced into the system at the proximal end of the delivery system between the inner canula and grey positioner ((**B**); white arrow) and advanced through the whole system until it becomes visible again ((**C**); white arrow) and passes through the rubber washer ((**C**); red arrow) in the grey handle. A standard 19 G puncture needle can be used to guide and externalize the guidewire out of the distal end of the grey handle ((**D**); white arrow). The rubber washer is advanced back into its original position to prevent leakage using forceps if it has been dislodged during guidewire passage (**E**). To facilitate removal of the guidewire during the procedure, a torque device can be added to enhance control (**F**).

Before a trigger wire is added, the grey inner positioner is completely removed from the Flexor introducer sheath by retracting it out of the Captor hemostatic valve. The rubber stop is removed from the grey handle (Figure 4A). Thereafter, a 0.018-inch guidewire (the authors use a V-18 ControlWire, Boston Scientific, Marlborough, MA, USA) is introduced into the grey positioner by introducing and advancing the back-end of the guidewire between the inner cannula and grey positioner (Figure 4B). The wire is advanced until it passes through the rubber washer in the exposed grey handle where the wire can be recovered with the help of a standard 19 G puncture needle (Figure 4C,D). The rubber washer is advanced back into position using forceps if it is dislodged by the guidewire (Figure 4E) to minimize leakage via the handle during the procedure. To facilitate removal of the preloaded wire during the implantation procedure, a torque device can be added to the back-end of the 0.018-inch guidewire just distal to the grey handle (Figure 4F). A maximum of three guidewires can be added one by one in this manner for use as a trigger wire and/or precannulated wires through selected fenestrations.

### 2.5. Construction and Engagement of the Diameter-Reducing Ties

**Figure 5:**
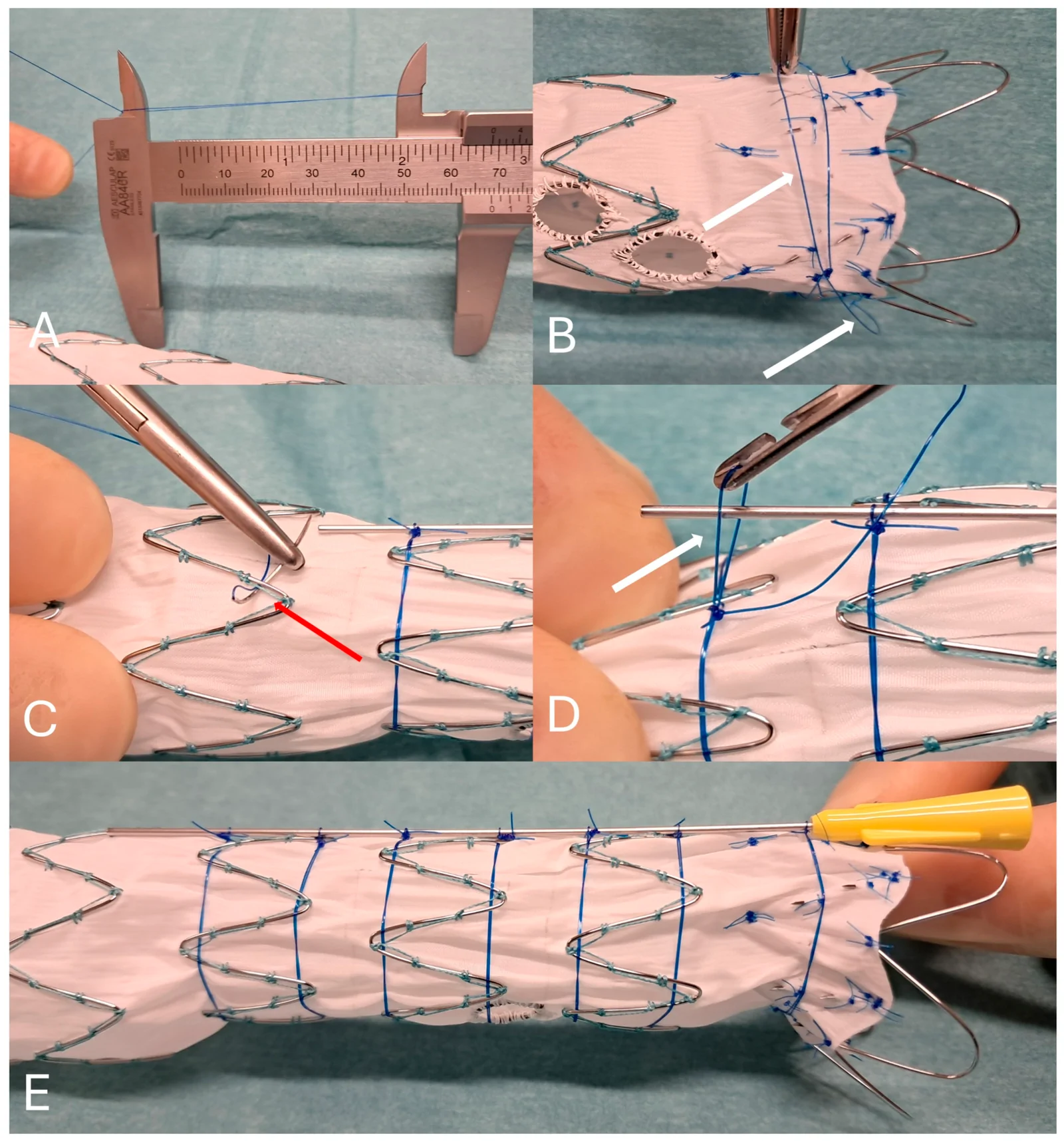
Circular diameter-reducing tie (DRT) loops can be created using vernier callipers set to the length corresponding to the circumference of the desired reduced diameter (**A**). The circular DRT at the level of the proximal sealing stent is attached to the fabric anteriorly using Prolene 6-0, creating two equal parts of the loop ((**B**); white arrows). The DRTs at the level of the other struts are placed by passing the blunt end of the attached needle between alternate struts and fabric, allowing for the loops to be pulled around the endograft ((**C**); red arrow). The DRTs are engaged by pulling the end of the loop without the knot through the end with the knot, whereafter an insertion tool can be used to temporarily secure the DRTs ((**D**); white arrow). Start by securing the DRTs at the proximal end of the endograft and work in a distal direction until all struts that require reduction have engaged DRTs (**E**).

Diameter-reducing ties (DRT) can be added in different ways. The authors prefer to use non-perforating circular DRT loops with two DRTs per stent-strut, allowing for uniform diameter reduction. The loops are created using Prolene 4-0 suture (Ethicon Inc., Somerville, NJ, USA) and with the help of vernier callipers (Figure 5A). The vernier callipers are set to the length that corresponds to the circumference of the desired reduced diameter. The needle is left attached to the loop. The DRT at the proximal sealing stent is attached to the fabric in the middle of the struts using Prolene 6-0 (Figure 5B), creating two equal loops. All other DRTs are placed by passing the blunt end of the needle between alternate struts and the endograft fabric and pulling the Prolene suture loop through this space (Figure 5C). To engage the DRTs, a Rumel tourniquet hook is used to pull the end of the loop without a knot through the other loop end at the posterior aspect of the endograft and to pass an insertion tool (Avenue Insertion Tool, Medline Int., Chateaubriant, France) through this loop, thereby restraining the DRT and allowing for the passage of the 0.018-inch guidewire wire later on (Figure 5D). The excess suture material and needle are removed close to the knot. Umbilical tape can be used to temporarily reduce the stent struts to facilitate the engagement of the DRTs. This process should be started at the proximal end of the endograft and should proceed distally until all struts that require reduction have engaged DRTs (Figure 5E) to allow for the removal of the insertion after the 0.018-inch guidewire has been passed through it. Alternatively, non-perforating circular DRTs that require perioperative rupturing with a moulding balloon can be used [17].

### 2.6. Remounting the Endograft on the Delivery System

**Figure 6:**
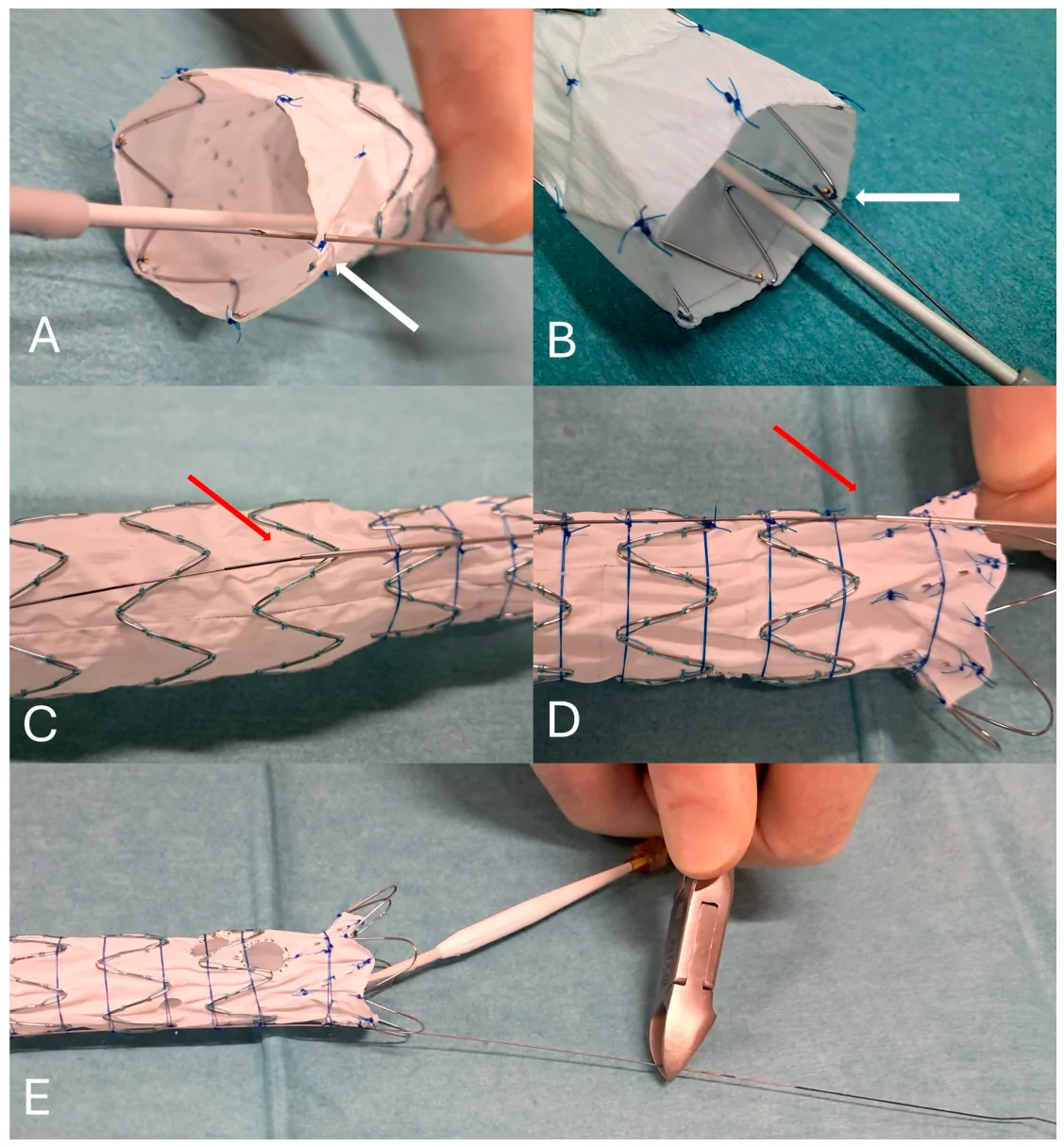
After the prepared modified endograft is placed over the delivery system, the 0.018-inch guidewire, with the help of a 21 G puncture needle, is passed from the inside to the outside of the endograft through the fabric in a strut valley of the most distal stent ((**A**,**B**); white arrows). The guidewire can then be advanced through the insertion tool ((**C**); red arrow). As the insertion tool is retracted in a proximal direction, the guidewire secures the DRTs ((**D**); red arrow). Lastly, the flexible end of the guidewire is cut with a wire cutter (**E**).

The authors recommend that the yellow-hubbed inner stylet is reinserted to straighten the proximal end of the delivery system to facilitate remounting of the endograft. To remount the endograft on the delivery system, the modified endograft with engaged DRTs is placed over the inner shaft. The 0.018-inch guidewire, to be used as a trigger wire, is then passed from the inside of the endograft through the fabric in a strut valley of the most distal stent to the outside using a 21 G puncture needle, whereafter it is passed through the insertion tool and DRTs (Figure 6A–C). The insertion tool can now be retracted leaving the DRTs engaged by the preloaded wire (Figure 6D). At the proximal end, the 0.018-inch guidewire is passed from the outside to inside of the endograft through the fabric at a strut peak and the flexible end of the guidewire is removed (Figure 6E).

**Figure 7:**
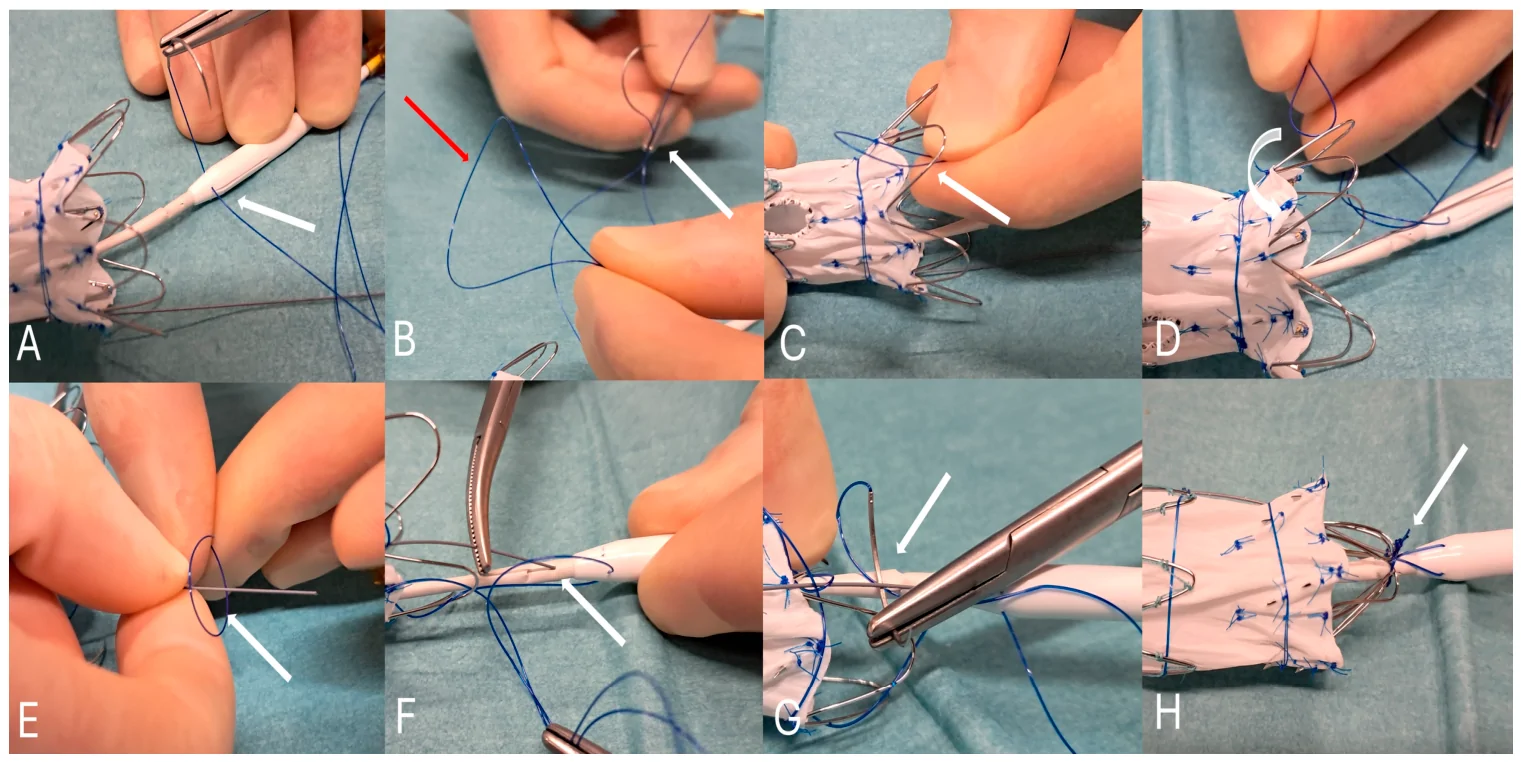
To re-attach the proximal bare alignment stent, a Prolene 2-0 suture is placed through the dilator tip just distal to the dilator tips’ maximum diameter ((**A**); white arrow). Clamps are placed on the suture ends ((**B**); white arrow), creating a loop ((**B**); red arrow). The looped end of the suture is passed through the five bare stents in a weaving in-out-out-in fashion ((**C**,**D**); white arrows). When the loop has passed through all the struts, the guidewire is placed through the loop ((**E**); white arrow) and into the dilator tip in the position of one of the original release wires ((**F**); white arrow). The blunt end of the needle is used to pass one of the ends of the suture between the guidewire and dilator tip (white arrow), thereby creating a second loop around the guidewire (**G**). Finally, traction is applied to both suture ends to restrain the bare stents and after which the suture is tied ((**H**); white arrow).

The proximal bare alignment stent is reengaged and secured to the dilator tip. To do this, a 2-0 Prolene suture (with a single needle) is placed through the dilator tip, and both ends of the suture are fixated by a clamp to form a loop (Figure 7A,B). The loop is then weaved through each of the bare struts (Figure 7C,D), alternately passing in-out out-in through the struts. After the loop has passed through all the bare struts, the 0.018-inch guidewire is passed through the suture loop and secured in the dilator tip in the position of one of the original release wires (Figure 7E,F). Lastly, the suture needle is passed between the trigger wire and dilator tip, and traction is applied to both ends of the suture to collapse the bare alignment stent and then tied to secure the bare alignment stent to the delivery system (Figure 7G,H).

**Figure 8:**
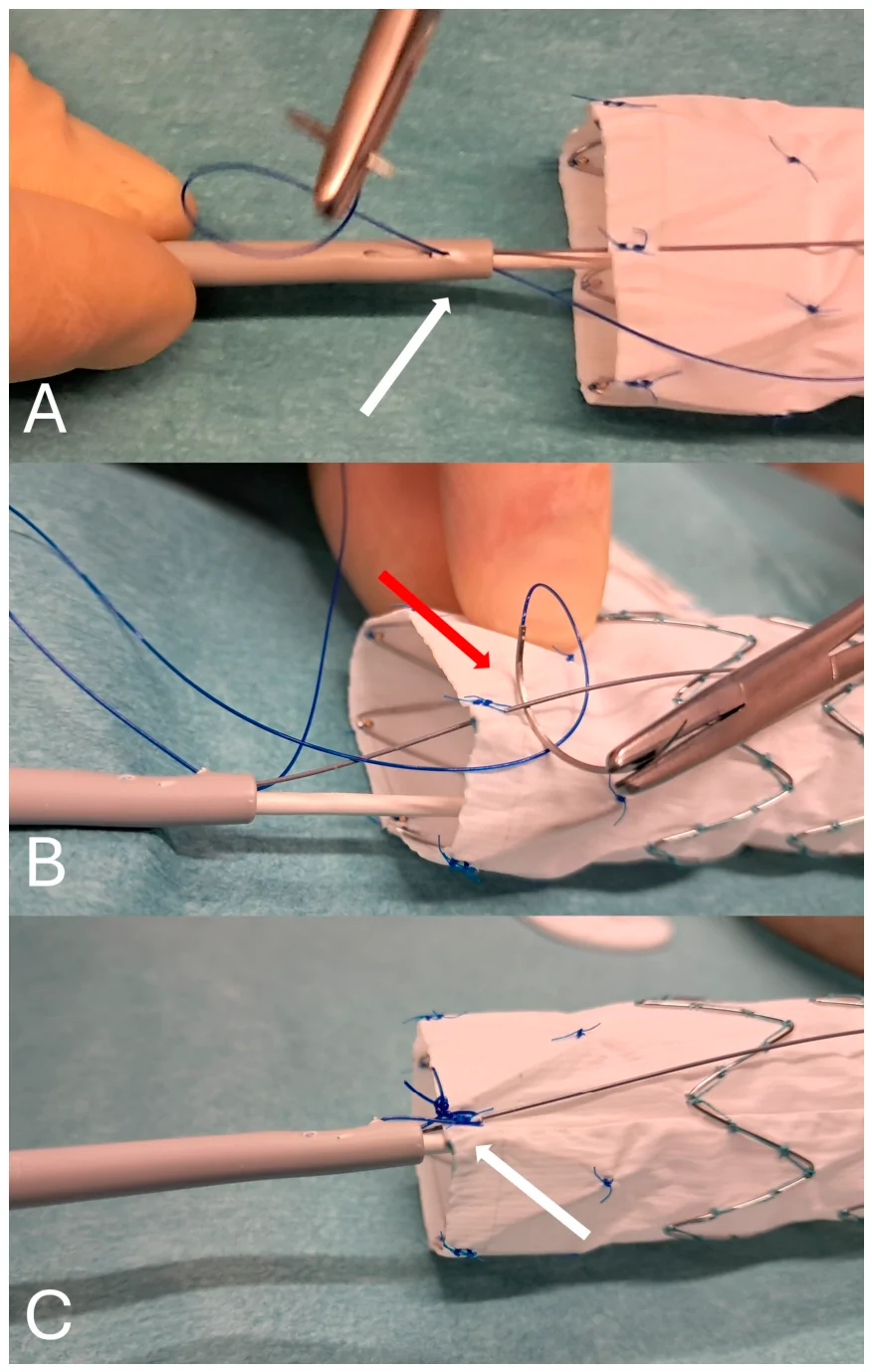
To secure the distal end of the endograft to the delivery system, a Prolene 2-0 suture is placed through the edge of the grey positioner ((**A**); white arrow). The suture needle is then passed between the guidewire and fabric, creating a loop around the wire ((**B**); red arrow). The suture is then tied ((**C**); white arrow) thereby both stretching the endograft to its intended length and fixing it to the delivery system until removal of the preloaded (trigger) wire.

The distal end of the endograft is then secured to the delivery system by placing a Prolene 2-0 suture through the grey positioner and passing the back end of the needle between the trigger wire and fabric (Figure 8A). The suture is not passed through the fabric or around a strut (Figure 8B). The suture is then tied, ensuring that the endograft is both expanded to its intended length and fixed to the delivery system until the trigger wire has been removed, allowing for optimal control during deployment and potential adjustments during implantation (Figure 8C). Proximal and distal fixation of the endograft to the delivery system in the described manner is required regardless of the type of DRTs used, as it allows for longitudinal and rotational control of the endograft during the procedure.

### 2.7. Resheathing the Endograft

**Figure 9:**
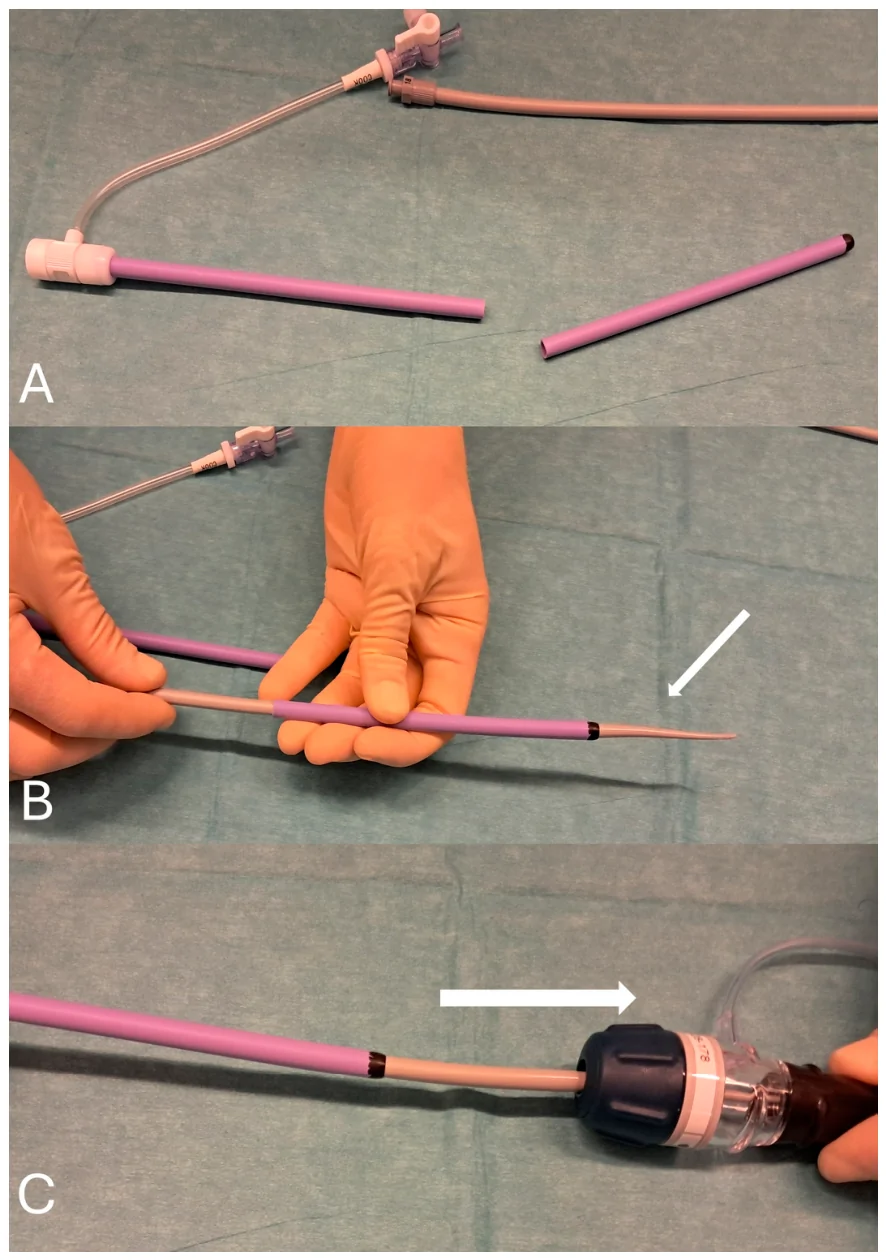
The distal tip of an introducer sheath of the same French size as the Flexor sheath is removed (**A**) and mounted on to its dilator ((**B**); white arrow). Place the dilator through the Captor hemostatic valve and advance the cutoff introducer sheath tip ((**C**); white arrow).

**Figure 10:**
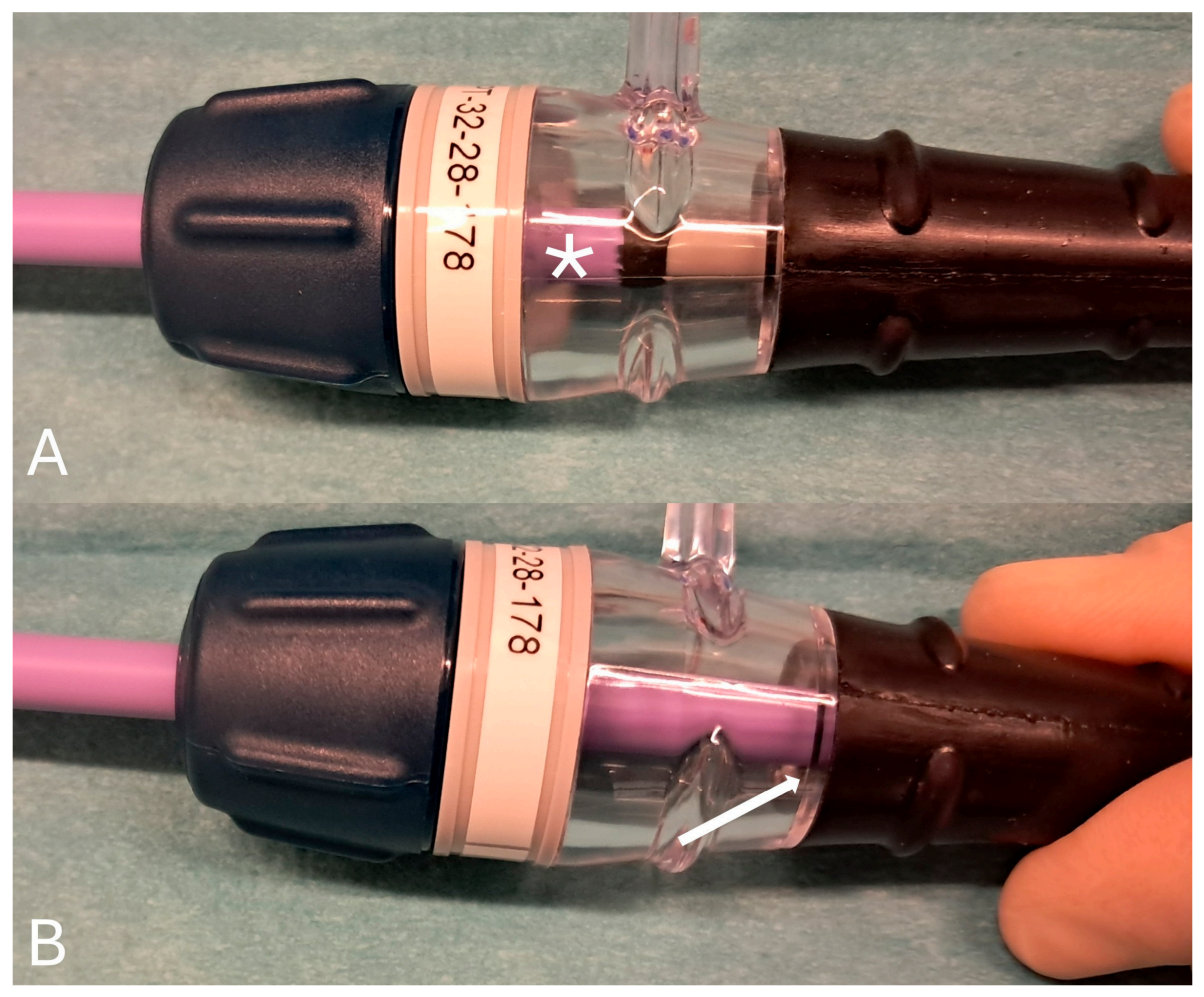
The cutoff introducer sheath tip will become visible within the Captor hemostatic valve (**A**); *) and should be advanced all the way in until it meets the proximal opening of the Flexor introducer sheath ((**B**); white arrow).

**Figure 11:**
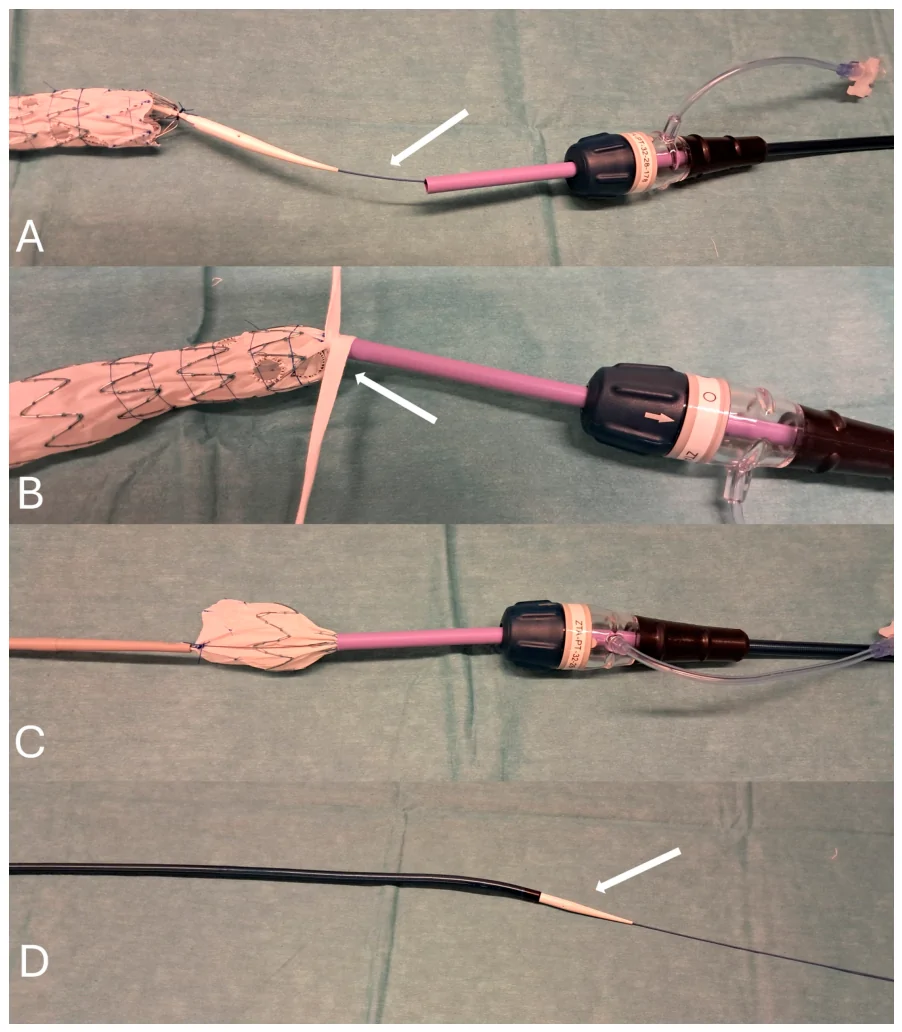
An Amplatz superstiff guidewire is passed through both the delivery system and Flexor introducer sheath ((**A**); white arrow)). Umbilical tape can be used to constrain the endograft ((**B**); white arrow)) whilst the delivery system and endograft are advanced through the cutoff introducer sheath and Captor hemostastic valve into the Flexor introducer sheath (**C**) until the delivery system dilator tip is back in its original position ((**D**); white arrow).

The endograft is then resheathed via the distal end of the delivery sheath, through the Captor hemostatic valve. For the endograft to pass through the Captor hemostatic valve into the Flexor introducer sheath, approximately 9 cm of the tip of a large-bore introducer sheath (same French size as the introducer sheath of the Alpha) is cut off and placed through the Captor hemostatic valve until it comes into contact with the sheath (Figure 9 and Figure 10). After removal of the yellow-hubbed inner stylet, a 0.035 Amplatz super stiff guidewire (Boston Scientific Corporation, Marlborough, MA, USA) is placed through both the shaft of the delivery system and introducer sheath (Figure 11A). Clamps can be placed at both ends of the Amplatz guidewire if needed to apply traction when resheathing. The endograft is then compressed using umbilical tape and introduced into the cut-off large-bore introducer sheath through the Captor hemostatic valve and into the Flexor introducer sheath (Figure 11B,C). Once the entire endograft is in the Flexor introducer sheath, the grey positioner is pushed forward until the dilator tip returns to its starting position outside the proximal end of the Flexor introducer sheath (Figure 11D). The cutoff large-bore introducer sheath tip needs to be fully removed from the delivery system by retracting it out of the Captor hemostatic valve and cutting it lengthwise. Failure to do this will prohibit the Flexor introducer sheath from being fully retracted, potentially preventing complete deployment of the endograft.

**Figure 12:**
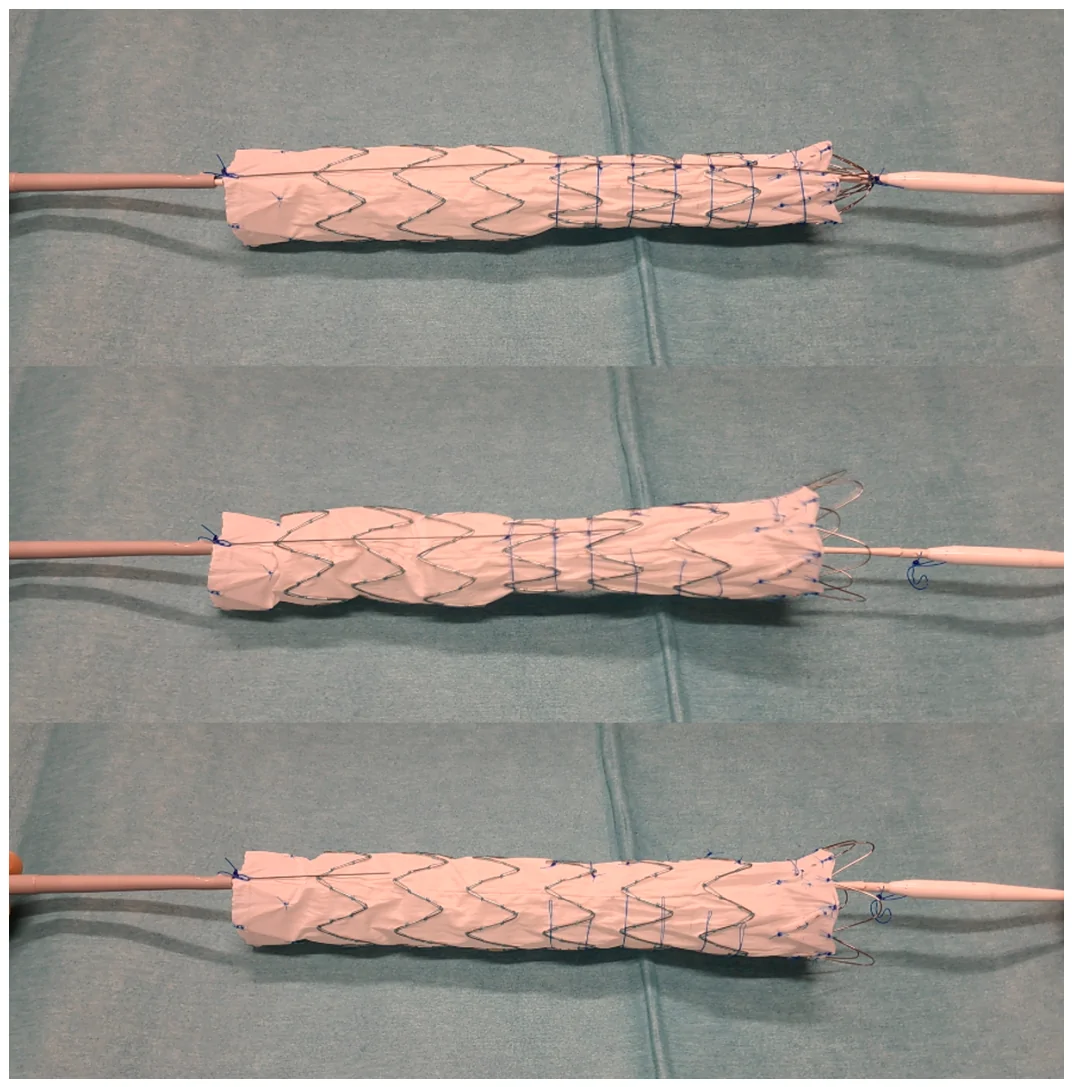
After endograft deployment, retraction of the guidewire (trigger wire) will release the bare alignment stent, followed by sequentially releasing the DRTs.

Now the procedure can be performed as a fenestrated endovascular aortic repair (FEVAR). After securing the target vessels the trigger wire can be retracted to fully release the endograft from the delivery system and at the same time release the bare alignment stent and disengage the DRTs (Figure 12). Rotating the trigger wire while it is being retracted can help to minimize friction between the trigger wire and DRTs, thereby reducing the downward pulling force on the endograft after the proximal attachment has been released.

**Figure 13:**
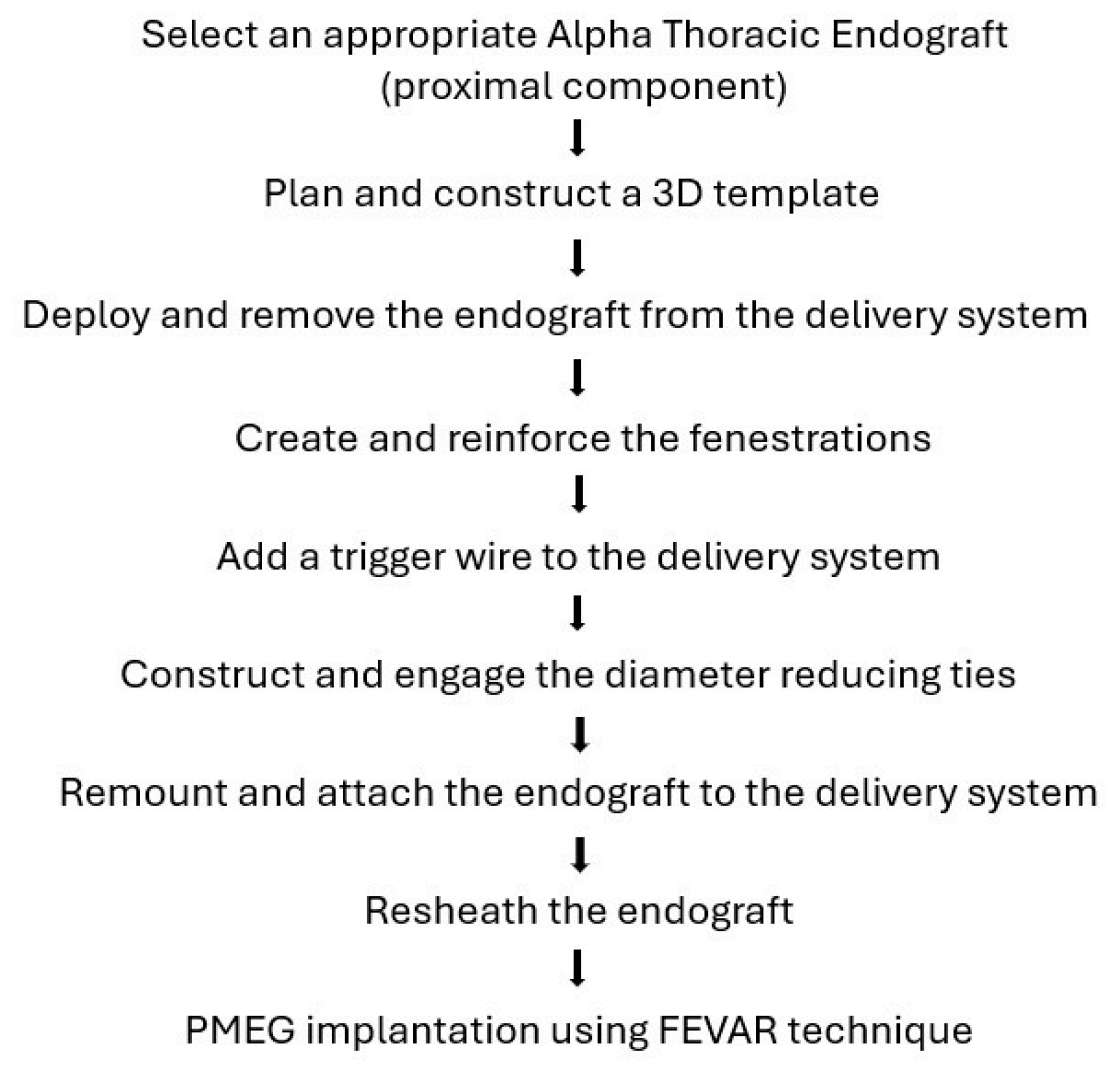
Summary of the steps required to construct a PMEG.

The workflow is summarized in Figure 13.

## 3. Discussion

The current article describes a step-by-step guide to constructing a PMEG for cAAA endovascular repair using the Zenith Alpha Thoracic Endovascular Graft platform while preserving the active fixation barbs. The Zenith Alpha Thoracic Endovascular Graft is one of the most commonly used platforms for performing PMEGs [4]. This is mainly due to the relatively large fabric surface area between the stent struts, which makes it more likely that a position can be achieved for all fenestrations without overlapping struts [9,12]. For endograft platforms where the struts are narrower and closer to each other, fenestration–strut conflicts during PMEG are more common and can require the modification of the struts or adjusting the placement of a fenestration [6,11]. The latter might, as a consequence, change the relative position of the fenestration to the target vessel, increasing the risk of misalignment. Misalignment between a fenestration and its target vessel is known to negatively impact target vessel stability in cAAA endovascular repair [14,19,20]. Although these situations are both undesirable and simultaneously more likely to occur for endograft platforms with narrower struts, such platforms are still frequently used. For reference, in the largest published series on PMEG to date by Tsilimparis et al., more than half (53%) of the 1274 included patients were treated with a Zenith TX2-based PMEG, which has relatively narrow struts compared to most other endograft platforms [4]. One of the main reasons for using a Zenith TX2 Dissection Endovascular Graft over the Zenith Alpha Thoracic Endovascular Graft would be that it is more easily resheathed [13]. The Zenith TX2 Dissection Endovascular Graft does not have active fixation barbs and can be removed from the distal aspect of the delivery system through the peel-away sheath, and then resheathed again through the same peel-away sheath. The Zenith Alpha Thoracic Endovascular Graft on the other hand has no peel-away sheath and is typically desheathed from the proximal aspect, and then resheathed against the direction of the proximal barbs [11,12,13]. To achieve this, the barbs must be removed. This requires cutting each barb, risking damage to the endograft fabric, or bending each barb until the metal breaks, which is more time consuming. However, with both methods, the remnant of the barb is not completely flush with the endograft fabric, resulting in a difficult resheathing process where the remnants still have the potential to damage the Flexor introducer sheath.

The method described in the current article was developed and standardized in an experienced PMEG centre as an evolution of previously used techniques. Using the current method, a bespoke endograft can be created in approximately 90 min (excluding planning) but total modification time can vary depending on the complexity of the required PMEG (e.g., number of fenestrations, DRTs and added wires).

The current article aims to contribute to greater procedural standardization by providing a detailed, reproducible step-by-step description of one modification technique. However, its reproducibility, learning curve, and performance compared to alternative techniques require further evaluation in future clinical and bench studies.

The current method allows for the preservation of the active fixation barbs and keeps the integrity of the endograft intact. Active fixation barbs are designed to enhance proximal attachment of the endograft to the aortic wall and are intended to contribute to endograft fixation. Retaining the barbs preserves the fixation mechanism of the device and may theoretically reduce migration and proximal seal failure [12]. However, there is limited direct evidence comparing outcomes between PMEGs with retained versus removed fixation barbs, and the decision remains largely operator dependent. Further clinical studies are needed to confirm the benefit of retaining the active fixation barbs.

It should be noted that the technique in the current article constitutes a deviation from the instructions for use (IFU) of the endograft used. This is the case for all PMEG modification techniques. Therefore, PMEG should be considered mainly as an alternative solution in urgent or time-sensitive settings when custom-made devices or suitable off-the-shelf branched endografts, which can perform well in selected urgent cases, are not available or not anatomically feasible [21,22,23,24]. When PMEG is used, the off-label nature places full responsibility on the physician. Obtaining patients’ informed consent and performing PMEG within a device exemption trial or after institutional approval in experienced aortic centres should be the norm [7].

The proposed technique requires complete removal, modification, remounting, and resheathing of the endograft and therefore demands meticulous attention during each step of the procedure. Potential technical pitfalls include damage to the endograft fabric, incomplete engagement or premature release of the diameter-reducing ties, and incorrect remounting of the delivery system. Although no specific safety concerns have been observed during the development of this technique, its off-label nature and technical complexity support restricting its use to experienced aortic centres familiar with PMEG construction.

Arguably, alterations to the endograft platform during PMEG should be minimized as all modifications made are inherently outside the intended use of the device and might come with unknown consequences. Off-the-shelf endografts undergo strict quality control, and deviation from the IFU through device modification may expose patients to increased risk. Removal of fixation barbs increases the complexity of the modification procedure and further alters the endograft, thereby compromising the integrity of the quality-controlled device. Following this line of thought, when faced with the choice to either remove the proximal fixation barbs, or to use a resheathing method that allows for their preservation, the latter might be viewed as preferential.

Although outcomes achieved with PMEG in cAAA endovascular repair are promising, an important limitation to appraising the available evidence on PMEG is the large number of different techniques used [4,8]. Future work should focus not only on prospective clinical and bench validation of this technique, but also on broader implementation of standardized PMEG workflows. Adoption of structured physician training and recently proposed guiding principles for PMEG construction may improve reproducibility, facilitate multicentre collaboration, and enhance the comparability of future clinical studies [25].

## 4. Conclusions

In conclusion, a PMEG can be constructed using the Zenith Alpha Thoracic Endovascular Graft platform while preserving the active fixation barbs using the current standardized modification protocol. The theoretical potential benefit to preserving the active fixation barbs needs to be confirmed in future clinical studies or bench testing, in addition to determining the reproducibility of the described technique and its comparative performance to alternative treatment options.

## Figures and Tables

**Table 1:** Suggested instruments, materials and sutures required to construct a PMEG.

Instruments	Materials	Sutures
Mayo Hegar needle holder	Cook Zenith Alpha Thoracic Endograft (proximal component)	Prolene
Forceps		2-0
Scissors	Introducer sheath (same size as endograft delivery system)	4-0
Scalpel, nr11 blade		6-0
Tweezers	Amplatzer SuperStiff guidewire 0.035 in/260 m	CV-6
Vernier callipers	V-18 ControlWire	
Rumel tourniquet hook	Endovascular snare	
Insertion tool	Sterile 3D template	
Wire cutter	Puncture needle: 19 G and 21 G	
High temperature cautery	Torque device	
	Cotton umbilical tape	

## Data Availability

No new data were created or analyzed in this study.

## Abbreviations

The following abbreviations are used in this manuscript:

PMEG	Physician-modified endografts
cAAA	Complex abdominal aortic aneurysms
CMD	Custom-made device
DRT	Diameter-reducing tie
ALMEG	Automated Laser Modification of the Endograft
FEVAR	Fenestrated endovascular aortic repair
IFU	Instructions for use
